# Enhancing crack detection and severity assessment in historical Tabiya basins using U-Net and adaptive thresholding

**DOI:** 10.3389/frai.2026.1741082

**Published:** 2026-02-27

**Authors:** Hafsa Matich, Jamal Attmani, Hajar Mousannif

**Affiliations:** 1 LISI Laboratory, Faculty of Sciences Semlalia, Cadi Ayyad University, Marrakesh, Morocco; 2 National School of Applied Sciences of Marrakech, Cadi Ayyad University, Marrakesh, Morocco

**Keywords:** crack detection, deep learning, fully convolutional network, heritage building, semantic segmentation, U-Net adaptive thresholding

## Abstract

The conservation of the historical Tabiya water basins remains paramount, with consideration being their cultural and architectural importance, though structural degeneration like surface cracking poses a formidable challenge to conservation work. Since the traditional methods of inspection are often subjective, tedious, and prone to error, these limitations are tackled in this study by means of presenting an automated system for surface crack detection and segmentation based on artificial intelligence and computer vision techniques. High-resolution images were captured on-site using a Canon EOS 1100D camera and analyzed within a comparative deep learning framework using four models, namely U-Net with MobileNetV2, ResNet-50, InceptionV3, and EfficientNetB7 backbones. The proposed system performs crack detection and segmentation, as well as quantitative measurements, including crack length, width, and severity assessment through skeletonization, a crack length estimation algorithm, and a crack width extraction method. Experimental results indicated that the MobileNetV2-based model outperformed all other tested architectures, with an accuracy of 98.7%, a recall of 98.2%, a precision of 99.1%, and an F1-score of 98.6%. Furthermore, the developed framework has also been deployed as a web application that allows users to upload or drag and drop images and select from four available models for automated analysis. This integrated system represents a strong, precise, and user-friendly tool for the digital preservation and structural monitoring of heritage water infrastructure.

## 1 Introduction

Heritage hydraulic structures are enormously significant cultural properties, yet their architectural properties suffer from material degradation with time. Surface cracking is one of the most frequently occurring and serious modes of distress in a structure. Cracks can form due to prolonged weathering, aging of the material, differential settlement, thermal variations, fluctuations in moisture, or seismic activity, and might be an early warning sign for progressive loss of structural integrity both in historic and modern constructions (Safiuddin et al., 2018; Qiu and Lau, 2023). In earthen and masonry heritage structures, even minor cracks can significantly accelerate deterioration by facilitating water ingress, promoting material disintegration, and ultimately compromising structural safety and long-term preservation (Iraniparast et al., 2023; Islam and Kim, 2019).

Crack assessment has traditionally relied on manual-based visual inspection, with rulers, crack gauges, or microscopes used to measure crack width, length, and evolution over a time duration (Yang et al., 2022; Yalew and Kim, 2023). Although widely practiced, such approaches are labor-intensive, time-consuming, and inherently subjective, particularly when applied to large-scale, irregular, or textured surfaces commonly encountered at heritage sites. Furthermore, variations in lighting conditions, surface complexity, accessibility constraints, and inspector expertise limit the repeatability, scalability, and objectivity of conventional inspection workflows (Ahila Priyadharshini et al., 2023; Fan et al., 2023; Al Biajawi et al., 2023; Alsharqawi et al., 2022; Candelaria and Kee, 2023; Cheng, 1997; Chang et al., 2019; Ding et al., 2023; Rahmati et al., 2023; Rasol et al., 2020). These limitations have driven growing interest in automated and data-driven alternatives for continuous and reliable structural monitoring.

Recent advances in computer vision and artificial intelligence have significantly transformed automated structural inspection. Image-based sensing combined with intelligent algorithms has enabled objective, repeatable, and data-driven condition assessments across a wide range of civil infrastructure applications (Kontoni et al., 2023; Koch et al., 2015; Chen et al., 2023). Within the broader field of structural health monitoring, AI-driven systems increasingly support resilient infrastructure management, smart city initiatives, and sustainable maintenance strategies (Raj et al., 2024; Kapoor et al., 2024). Comprehensive reviews consistently highlight the expanding role of deep learning techniques for detecting and characterizing damage in concrete and masonry structures (Hamidi et al., 2025).

Within this context, convolutional neural networks (CNNs) have demonstrated notable success in crack detection. Earlier CNN-oriented works concentrated mostly on picture-level classification, indicating whether cracks were present or not but providing no spatial localization of the cracks (Elhariri et al., 2022). While useful for coarse screening, such approaches are insufficient for structural diagnosis, where accurate quantification of crack geometry and severity is essential. To overcome this limitation, semantic segmentation architectures—most notably U-Net—have gained prominence due to their encoder–decoder design and skip connections, which enable precise pixel-wise delineation of crack patterns (Hacıefendioğlu et al., 2023).

It has been shown in recent advances that architectural innovations can considerably enhance the segmentation performance. Residual learning and feature fusion strategies in RS-Net have improved crack boundary clarity and severity estimation in pavement applications (Ali et al., 2024), while multiscale contextual integration in MSMC-U-Net has improved robustness under complex backgrounds and variable crack morphologies (Pervaiz et al., 2025). Comparative studies consistently show that U-Net-based architectures offer strong segmentation accuracy, but that performance is highly dependent on the choice of backbone network and the trade-off between accuracy and computational cost (Garg et al., 2025).

In the realm of crack detection, although CNN models have dominated, transformer models have gained traction due to their ability to model long-range dependencies. Hierarchical transformer architectures have demonstrated competitive performance on concrete and bituminous surfaces, particularly under challenging noise and texture conditions (Li et al., 2024). However, such models typically require substantial computational resources, limiting their suitability for real-time deployment or resource-constrained heritage monitoring scenarios.

Besides, heritage and cultural structures present extra challenges that do not exist in ordinary concrete infrastructure Variations in material composition, erosion-induced surface irregularities, heterogeneous textures, and uneven illumination significantly complicate crack detection and segmentation. Attention-enhanced U-Net models integrated with optical pulsed thermography have successfully revealed cracks on ancient murals, underscoring the potential of deep learning for heritage applications (Cui et al., 2024). Similarly, recent pixel-level segmentation studies on historic surfaces confirm the feasibility of deep learning approaches, while emphasizing the need for carefully curated datasets and tailored model designs (Söyleyman et al., 2022).

Despite these advances, a clear research gap remains. Existing studies predominantly focus on pavements, bridges, and modern concrete structures, with limited attention to heritage hydraulic structures composed of earthen and masonry materials, such as Tabiya water basins (Tran et al., 2024). Moreover, there is a lack of systematic comparison of U-Net variants with different transfer learning backbones under heritage-specific surface conditions, where crack patterns are subtle and closely intertwined with material heterogeneity.

**Figure 1:**
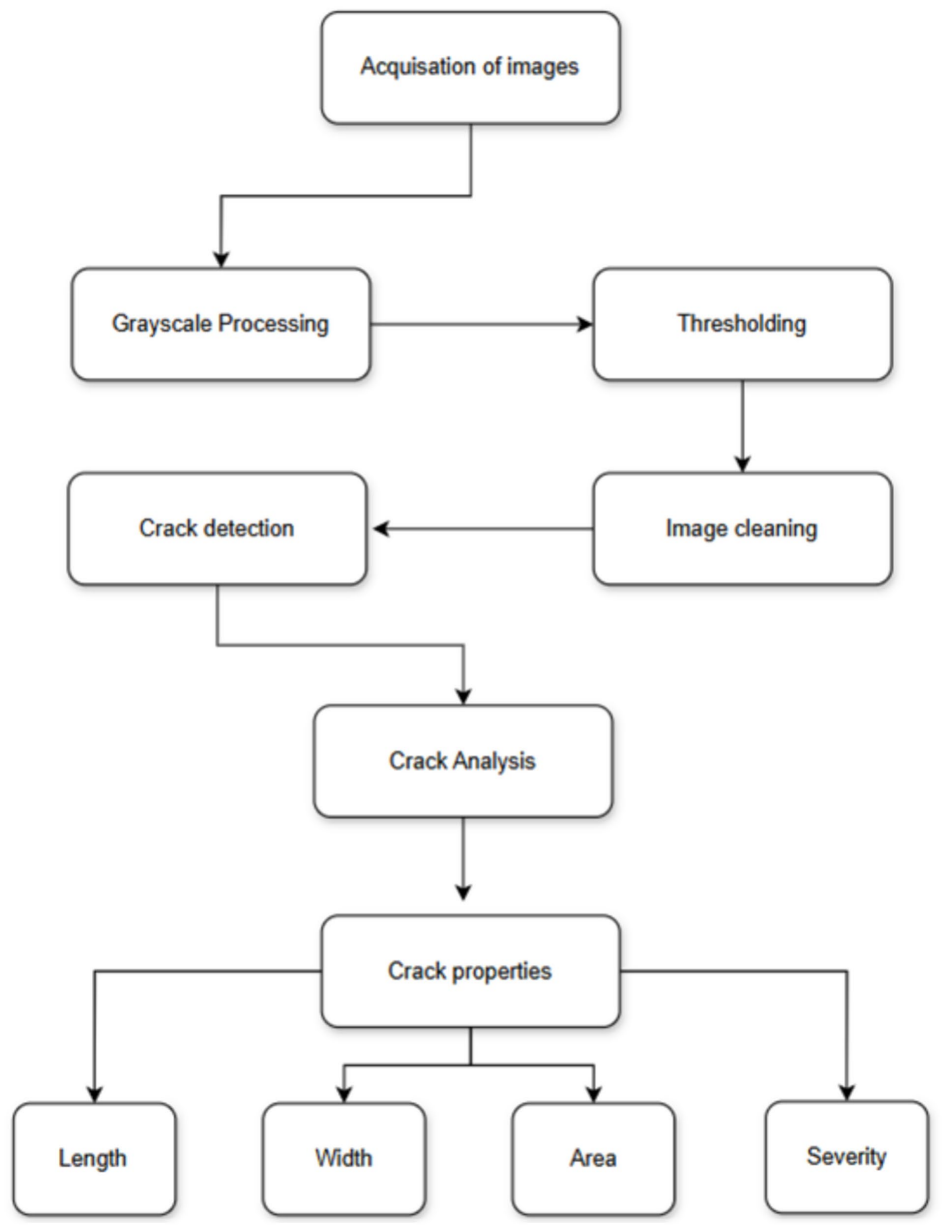
The proposed crack detection workflow.

Addressing this gap, the present study proposes an AI-driven workflow for automated detection, segmentation, and quantitative assessment of surface cracks in Tabiya water basins. Four U-Net variants incorporating different transfer learning backbones—MobileNetV2, InceptionV3, ResNet-50, and EfficientNetB7—are systematically evaluated in terms of segmentation accuracy, robustness, and computational efficiency on complex heritage surfaces. The proposed approach aims to deliver a scalable, objective, and practical tool to support heritage conservation and maintenance efforts. The overall workflow is illustrated in Figure 1.

## 2 Materials and methods

### 2.1 Case study description

This study is aimed at the automatic detection of surface cracking on ancient earthen structures, with Sahrij Labgar in the Kasbah district of Marrakech serving as a specific case for analysis. Constructed during the Almoravid period (Visconti and Capozzi, 2014), the reservoir of Sahrij Labgar (from the 11th to the 12th century) is an important element linking to the early hydraulic engineering system of the city. The basin has a close relationship with the *Khattaras*^1^, which form a network of underground galleries and storage basins devised for irrigation purposes. The basin assisted substantially in the irrigation of royal gardens and farming grounds and thus stands as a testimony to the highly advanced technical and environmental adaptations accomplished by Almoravid engineers.

Sahrij Labgar is architecturally representative of a large quadrangular reservoir, measuring approximately 3.8 m deep, and having storage of above 40,000 m^3^. The walls of the structure, estimated to be 2.3 m thick, were built using the traditional material of tâbiya (rammed earth), where earth-lime-straw-gravel mixtures are repeatedly laid to create big solid walls. The inner surface of this basin has then been treated with a lime-based plaster and tadelakt, which is a polished waterproof coating characterized by its durability and resilience toward water intrusion.

However, while the antiquity of these materials is enduring, the processes of deterioration greatly supported by aging, environmental exposure, and changing levels of moisture are always taking place and manifesting in observable effects: surface cracking, erosion, biological colonization, and partial detachment of coatings. The irregularities woven into the diverse pathologies therefore pose a genuine conservation challenge with respect to the heterogeneous area of earthen surfaces of the structure.

Thus, Sahrij Labgar has become an ideal test site for AI-enabled crack detection and automated defect mapping concerning heritage. The cases being considered are marked with historical significance, coupled with complex surface morphology and environmental vulnerability, with an aim to assess this case as a prominent one where the latest visual analysis might serve to reinforce non-invasive monitoring and preventive conservation strategies of traditional earthen hydraulic architecture.

### 2.2 Dataset description

**Figure 2:**
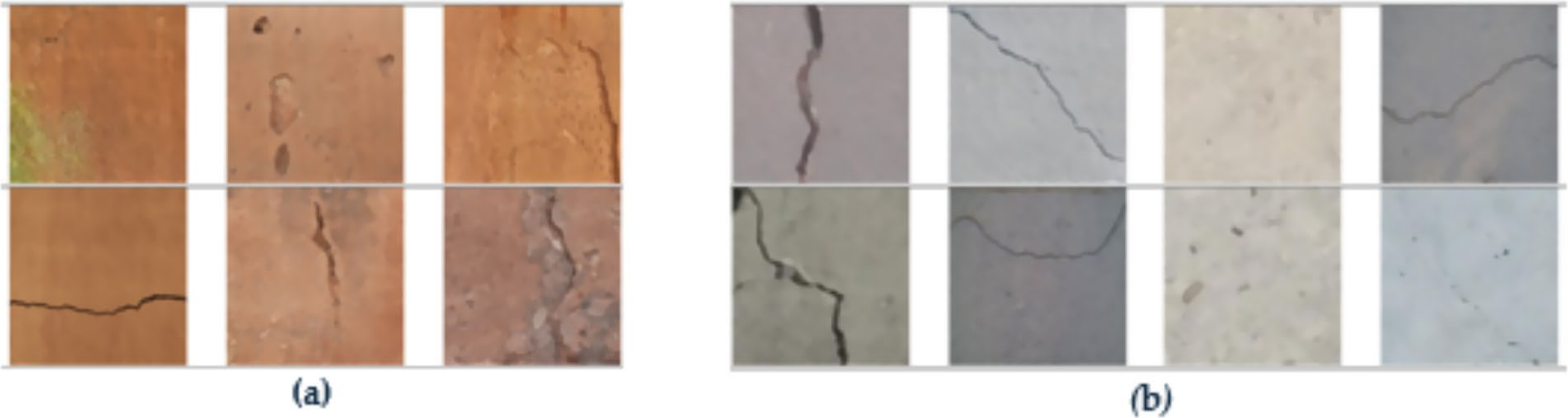
Training data examples from dual sources: **(a)** on-site collected image and **(b)** external database sample.

**Figure 3:**
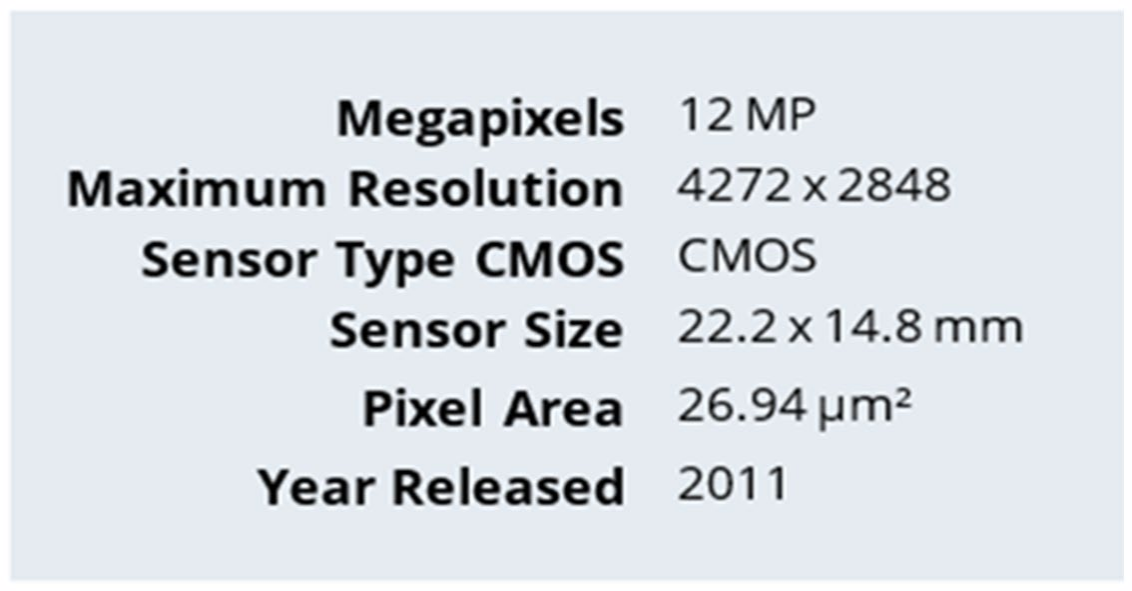
Canon EOS 1100D.

In this research, relevant open crack datasets were exploited, along with *in situ* image acquisition, the two being systematically merged to forge a large and diverse dataset targeting the training and evaluation of the segmentation model proposed. A total of 2,500 images of high quality (Figure 2a) were obtained *in situ* from the ancient Tabiya water basins using a Canon EOS 1100D DSLR camera (12.2 MP, 30 mm lens; Figure 3). Image acquisition was carried out under early morning natural light to avoid unwanted shadows or specular reflections, while a parallel movement acquisition method was employed. Approximately 50–60 images were taken per wall in so-called “overlapping” mode to ensure complete surface coverage. Of the acquired images, 1,625 showed visible cracks, while 875 showed intact concrete.

**Figure 4:**
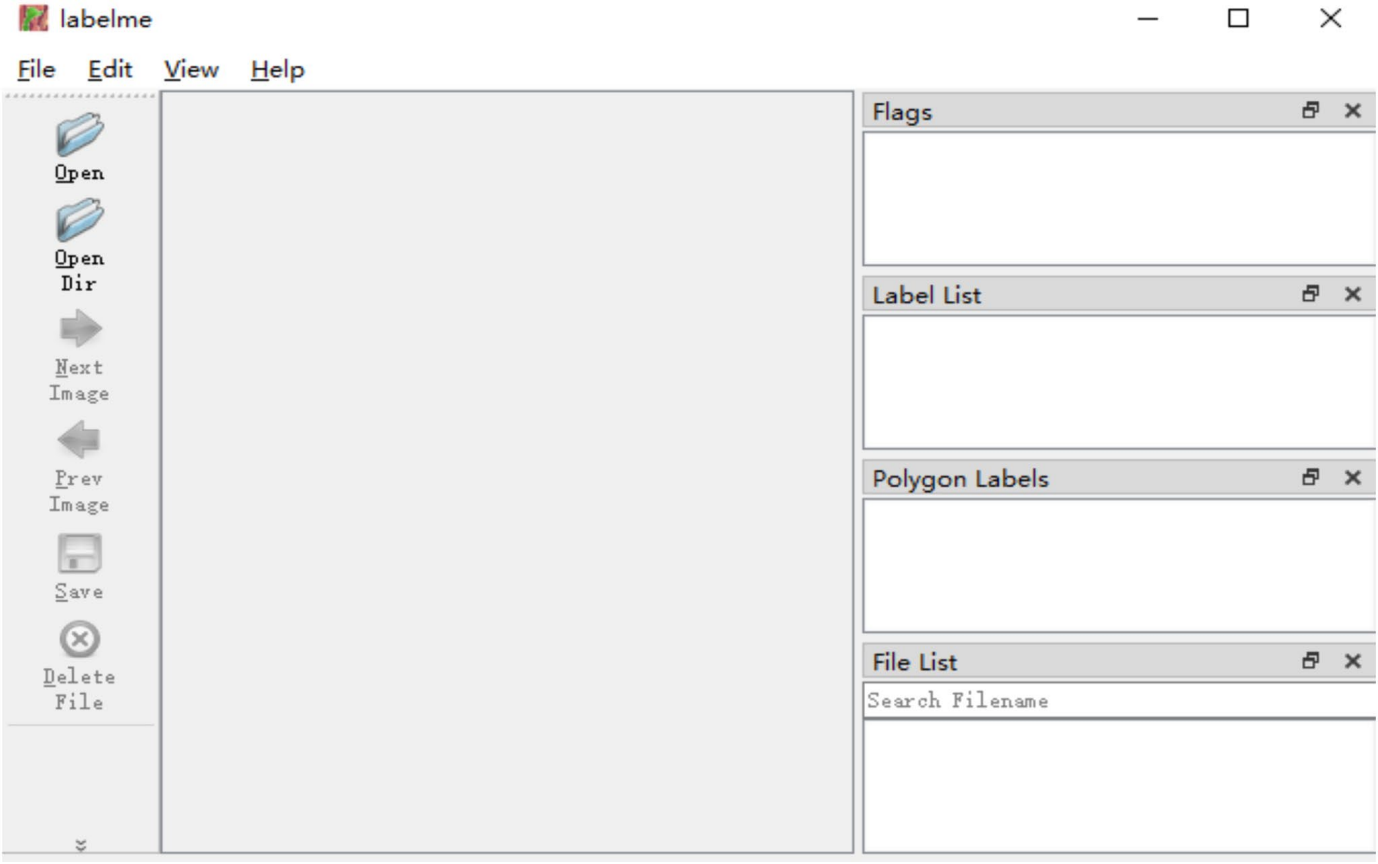
The main interface of LabelMe.

Ground-truth masks for these in situ images were generated through manual pixel-by-pixel annotation using the LabelMe tool (Figure 4). The crack regions were carefully delineated following a predetermined annotation protocol for uniformity in crack definitions and labeling accuracy across all heritage images (Russell et al., 2008; Wada, 2019).

To cope with the in-situ dataset’s limited size and enhance model generalization, the training data were supplemented with 7,500 annotated images extracted from two well-known public crack datasets (Figure 2b). The first dataset, SDNET2018, contains labeled crack and non-crack images collected from bridge decks, walls, and pavements, while the second dataset, CFD, offers manually annotated pixel-level crack masks for road-surface defects. Before integration, all external images and corresponding masks were normalized to the same resolution, while the differences in labeling conventions were reconciled by collectively adopting a binary crack-background representation. This ensured a uniform labeling rule across heritage and public datasets and allowed seamless joint training of the segmentation model.

The final combined dataset consists of 10,000 fully annotated images divided such that 8,000 images served for training (80%) and the remaining 2,000 images for testing (20%), with proportional contributions from both in-situ and public datasets in each subset. Such a proportional split would ensure that the heritage images maintain a significant proportion in the test set in order to directly and fairly assess the performance of the model in the prime application domain. This evaluation strategy would comply with the state-of-the-art practices in deep learning–based image segmentation, which, after all, maintains an independent test set for the reliable estimation of model generalization performance (Bishop, 2006; Géron, 2019). Very similar data-splitting strategies have been heavily relied upon in crack detection and structural inspection studies to guarantee balanced and unbiased evaluation under diverse surface conditions (Cha et al., 2017; Zou et al., 2019).

### 2.3 Preprocessing

**Figure 5:**
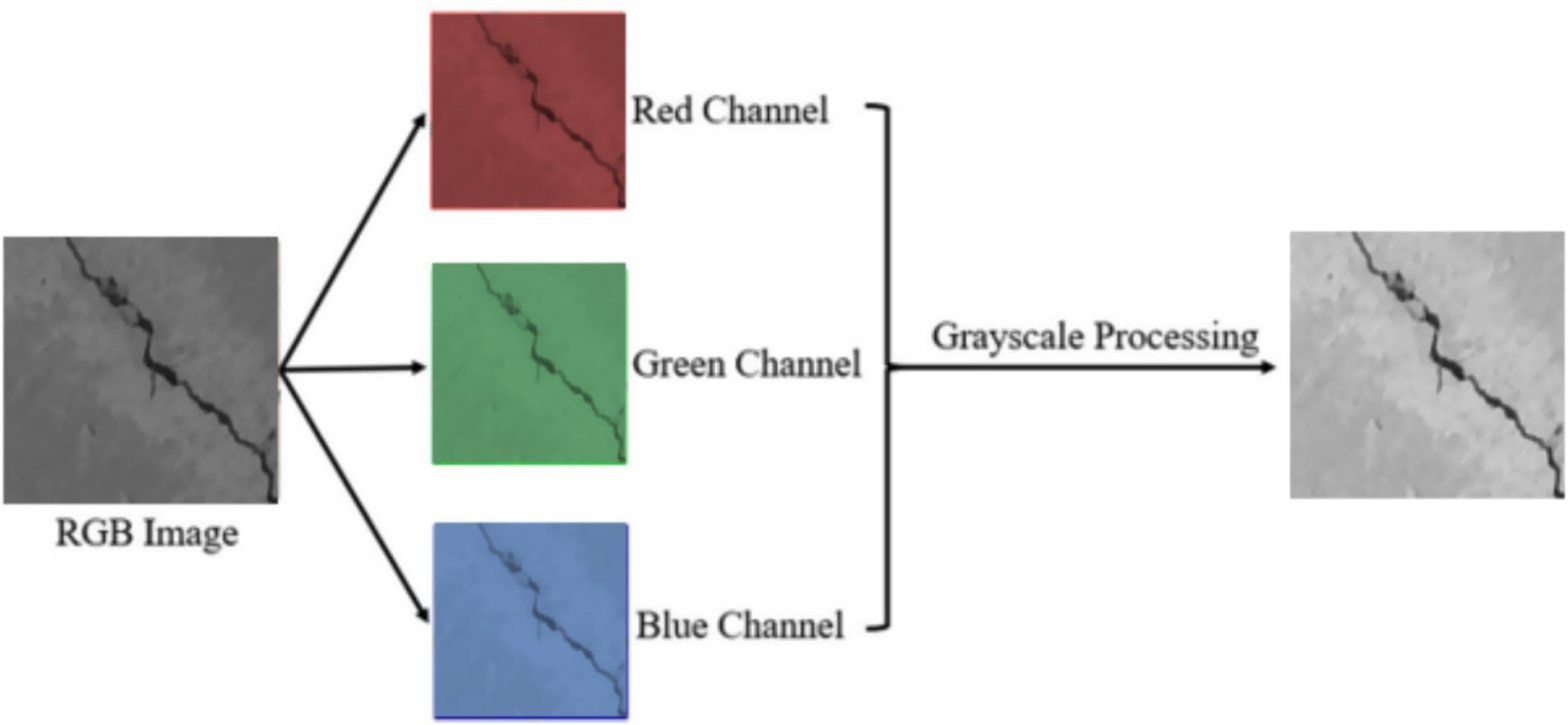
RGB-to-grayscale conversion.

Preprocessing is of prime importance and crucial component of crack segmentation pipelines that provides the model with standard, de-noised inputs and helps increase the stability and accurate prediction. The first preprocessing was grayscale conversion (Figure 5), which reduced the redundancy across channels and improved the structural contrast in the concrete texture allowing a segmentation model to concentrate on intensity-based crack patterns rather than differences in color (Gonzalez and Woods, 2007). Since concrete surfaces are affected mostly with shadows and stains with heterogeneous illumination, a dedicated thresholding step was introduced to model-specific enhancement the visibility of cracks prior to segmentation. Thresholding is a common practice for highlighting areas of low-intensity cracks while suppressing background noise in most related works, especially in classical computer vision workflows and hybrid deep learning pipelines, where illumination artifacts can often cover fine crack structures.

**Figure 6:**
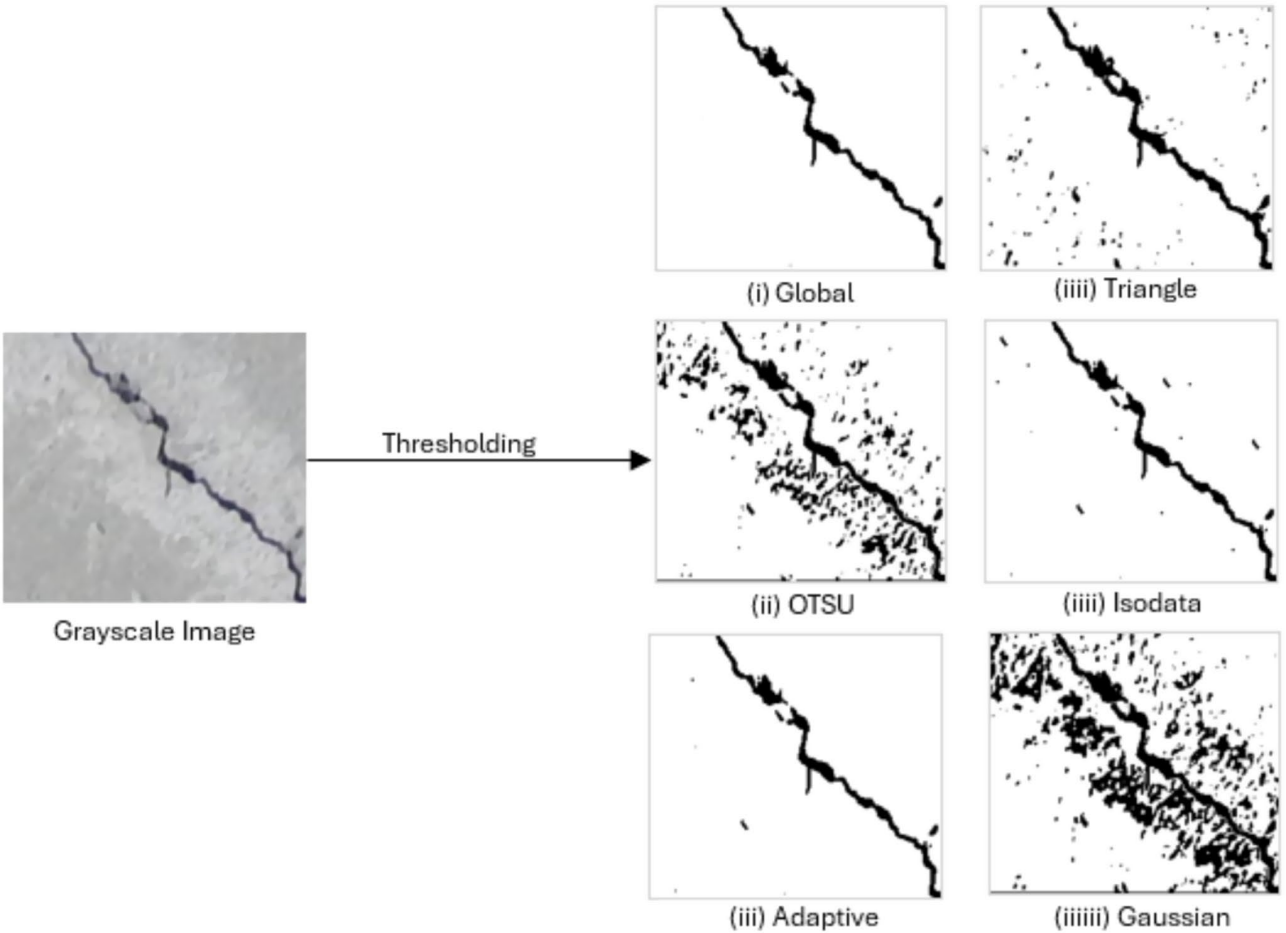
Comparison of image thresholding methods.

There were multiple thresholding techniques evaluated over which the ideal method should be selected to be used on the dataset, such as Global, Otsu, Triangle, Isodata, and Adaptive (Table 1). Using local pixel-neighborhood statistics to compute thresholds – Adaptive or local thresholding technique – proved to provide consistently better results. This locality-aware approach preserved thin and low-contrast cracks, maintained structural continuity, and reduced false positives caused by surface artifacts. It was particularly proved effective under uneven illumination and textured concrete backgrounds yielding the cleanest binary representations of cracks. On the basis of these observations and quantitative assessments, Adaptive thresholding was selected as the primary preprocessing method, providing high-quality input masks that greatly improved subsequent segmentation performance (Figure 6).

**Table 1:** Comparison of thresholding methods (Amiriebrahimabadi et al., 2024).

Method	Time	Sensitivity to noise
Global	Very fast	High is a simple threshold, amplifying noise in full image.
Otsu	Fast	Space is meant for clean bimodal images, but difficult to manage with noisy histograms.
Adaptive	Slower	Ignores local noise and illumination variations with effectiveness.
Triangle	Fast	medium is best for clean, sparse histograms; however, results can still be affected by noise.
Isodata	Medium	Medium- Some noise in the iterative process can average it out.
GMM	Slowest	Low: Models noise and complex distributions well.

### 2.4 Semantic segmentation models

There are many pre-trained convolutional neural networks available for classification tasks. What illustrates both the number of architectural possibilities offered to developers and their contrast includes accuracy, speed of prediction, and size of memory footprint. MobileNetV2 (Qayyum et al., 2022), EfficientNetB7 (Mazni et al., 2024), InceptionV3 (Qayyum et al., 2022), and ResNet-50 (Bussa and Boppana, 2025), whose distinctive reputation lies in weighing high accuracy against small model size, were selected (Table 2). To the machine learning community, these four models represent models with the lowest computational and memory efficiency. In several scenarios, practical usage together with limited resource allocated for computation and memory makes them attractive options. Table 1 presents typical features of such pre-trained model instances.

**Table 2:** Consideration of parameter counting and computational loads for assessing four eminent CNN architectures: MobileNetV2, InceptionV3, ResNet-50 and EfficientNetB7.

Model	Parameters (million)	Size (MB)
MobileNetV2	3.5	14
InceptionV3	23.9	92
ResNet-50	25.6	98
EfficientNetB7	66.7	256

Of course, MobileNetV2 does have some bragging rights with respect to fewer parameters and smaller size.

#### 2.4.1 U-Net

**Figure 7:**
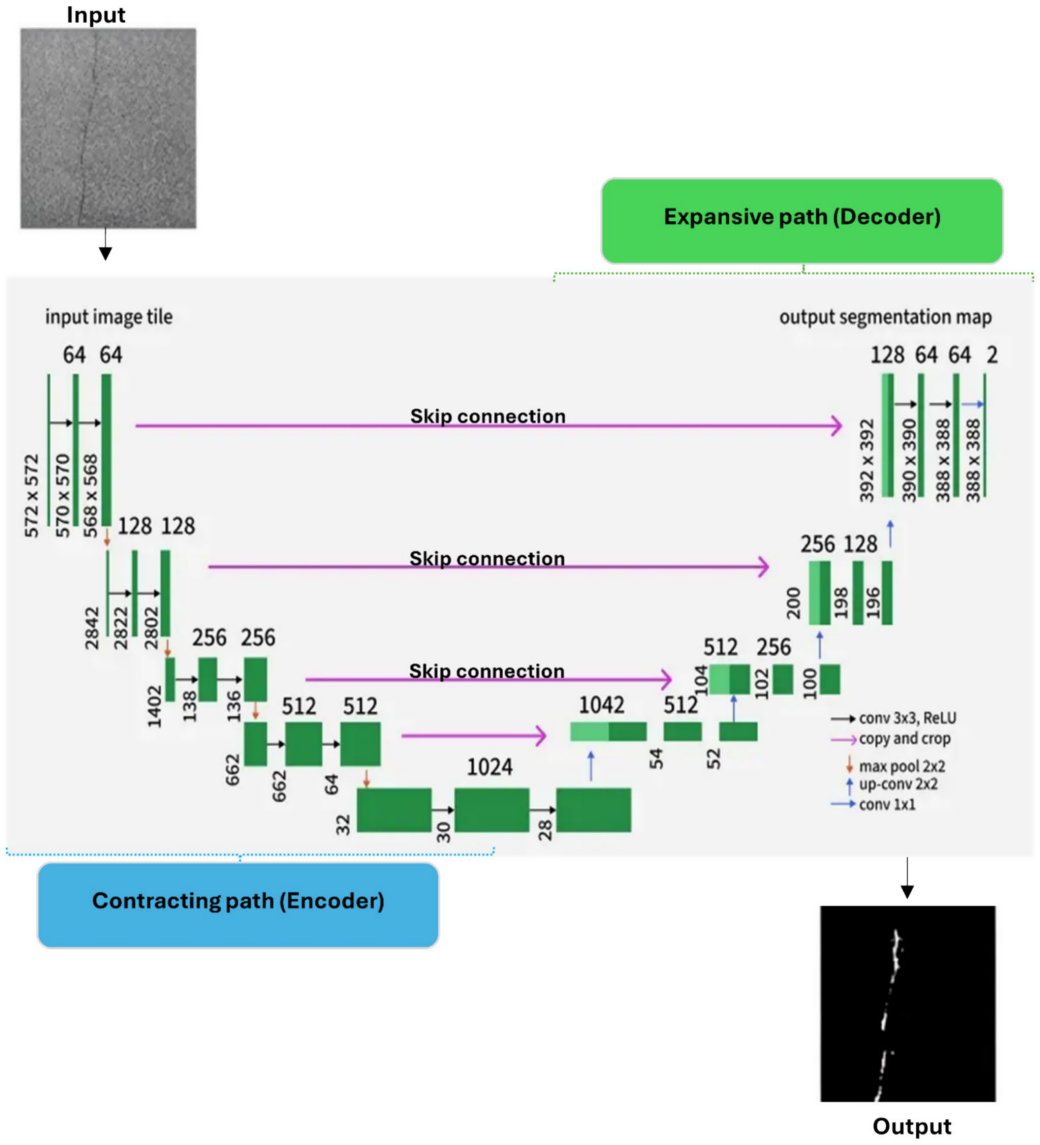
U-Net architecture illustrating the input image pipeline, encoder–decoder structure, and resulting segmentation map.

The U-Net architecture is formed by characteristic U-shape with the encoder-decoder design initially postulated by Ronneberger et al. (2015). Figure 7 illustrates the full process, including input image, U-Net architecture, and output segmentation mask. Among the most popular techniques for semantic segmentation, U-Net is particularly strong when training data are scarce.

U-Net architecture consists of a contracting path (encoder) that captures contextual information and an expansive path (decoder) that enables precise localization. These two paths are joined via a bottleneck (bridge). The contracting path follows the typical structure of convolutional neural networks, taking the input image through successive encoding blocks and extracting increasingly abstract feature representations.

In this study, the selected pre-trained CNN models are integrated into the U-Net framework as encoder backbones, replacing the standard convolutional encoder while retaining the original decoder structure. The encoder extracts multi-scale hierarchical features, which are progressively upsampled by the decoder to restore spatial resolution.

The segmentation mask for crack detection is generated from decoding along the expansive path. During decoding, feature maps are iteratively upsampled and concatenated to their subsequently downsampled counterparts from the encoder through skip connections. This allows the network to restore valuable spatial cues that may have been lost during downsampling, thus giving it the ability to accurately delineate fine crack structures.

Finally, through a 1 × 1 convolution and the sigmoid activation function, a binary segmentation mask is generated. Each pixel is classified as either “crack” or “non-crack,” enabling precise pixel-level crack segmentation by combining global contextual information with fine spatial details.

#### 2.4.2 U-Net-MobileNetV2

**Figure 8:**
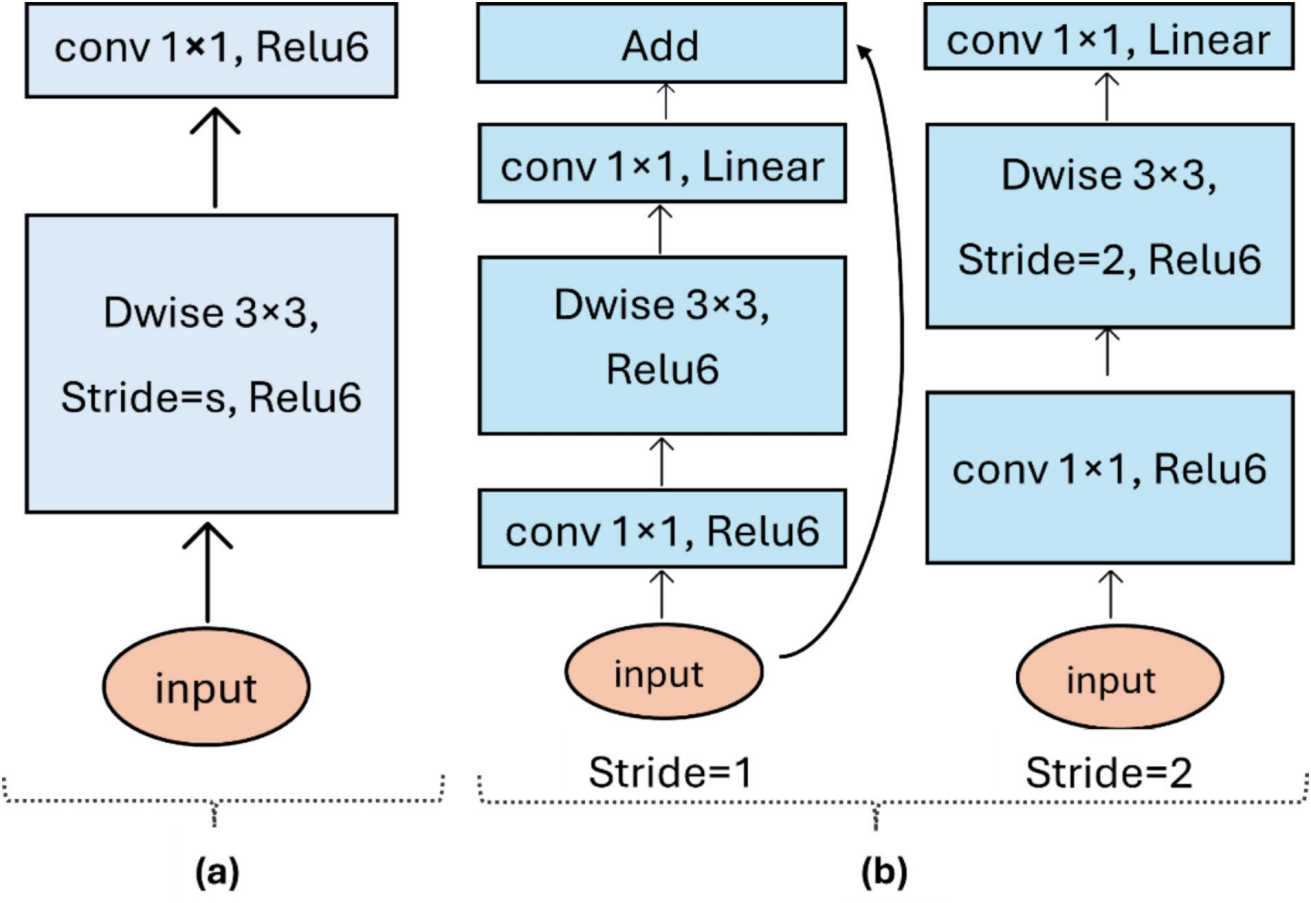
Architecture variants: **(a)** U-Net-MobileNet and **(b)** U-Net-MobileNet-V2.

A hybrid model is thus developed where the original U-Net encoder is replaced with a pre-trained MobileNetV2 backbone (Figure 8b), in contrast to the earlier U-Net-MobileNet variant (Figure 8a). This integration allows MobileNetV2 to serve as the feature-extraction encoder while retaining the original U-Net decoder with skip connections. Transfer learning accelerates feature extraction and improves performance, particularly on the limited in-situ dataset (Qayyum et al., 2022). The decoder is similar to that of the U-Net, employing transposed convolutions with skip connections to the MobileNetV2 feature maps. This reduces the total number of trainable parameters and improves convergence.

#### 2.4.3 ResNet-50

**Figure 9:**
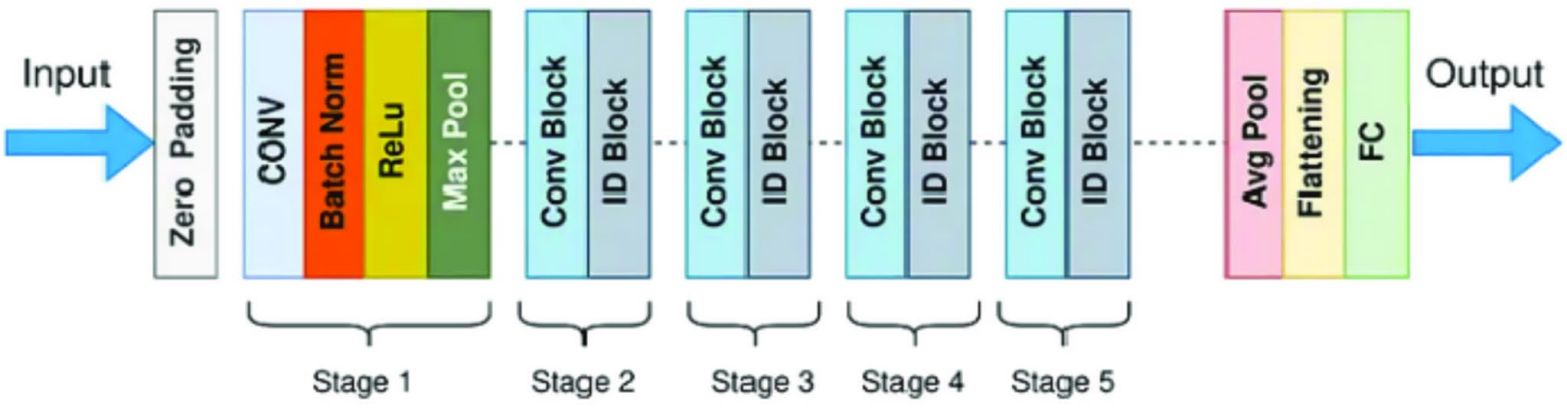
ResNet-50 model architecture.

The ResNet-50 architecture (Figure 9) was adapted as a U-Net encoder. The input layer was modified to accept 320 × 320 grayscale images, and the encoder’s residual blocks extract hierarchical features useful for crack segmentation. The original fully connected classification head is removed, and the decoder of the U-Net reconstructs the segmentation mask from these features. The deep residual blocks are features of the network that allow effective training through skip connections and learn robust features for distinguishing crack patterns (Bussa and Boppana, 2025).

#### 2.4.4 InceptionV3

**Figure 10:**
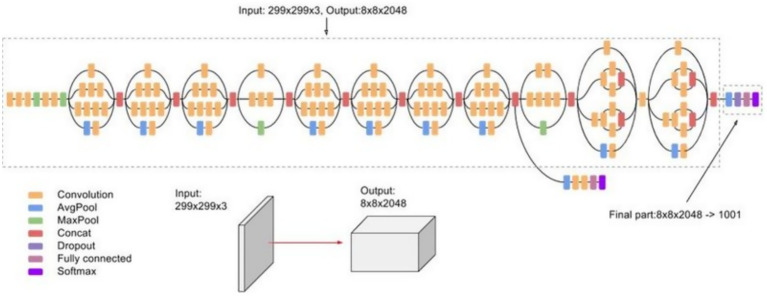
InceptionV3 model architecture.

The InceptionV3 model (Figure 10) was employed as the U-Net encoder, leveraging its multi-scale feature extraction capability. The classification head was replaced with a global average pooling layer, feeding features to the U-Net decoder for segmentation (Qayyum et al., 2022). Data augmentation techniques (such as rotation or brightness adjustment) were also applied during training for robustness. The parallel convolutional paths with varying kernel sizes enable the encoder to capture crack patterns at different widths and orientations.

#### 2.4.5 EfficientNetB7

**Figure 11:**
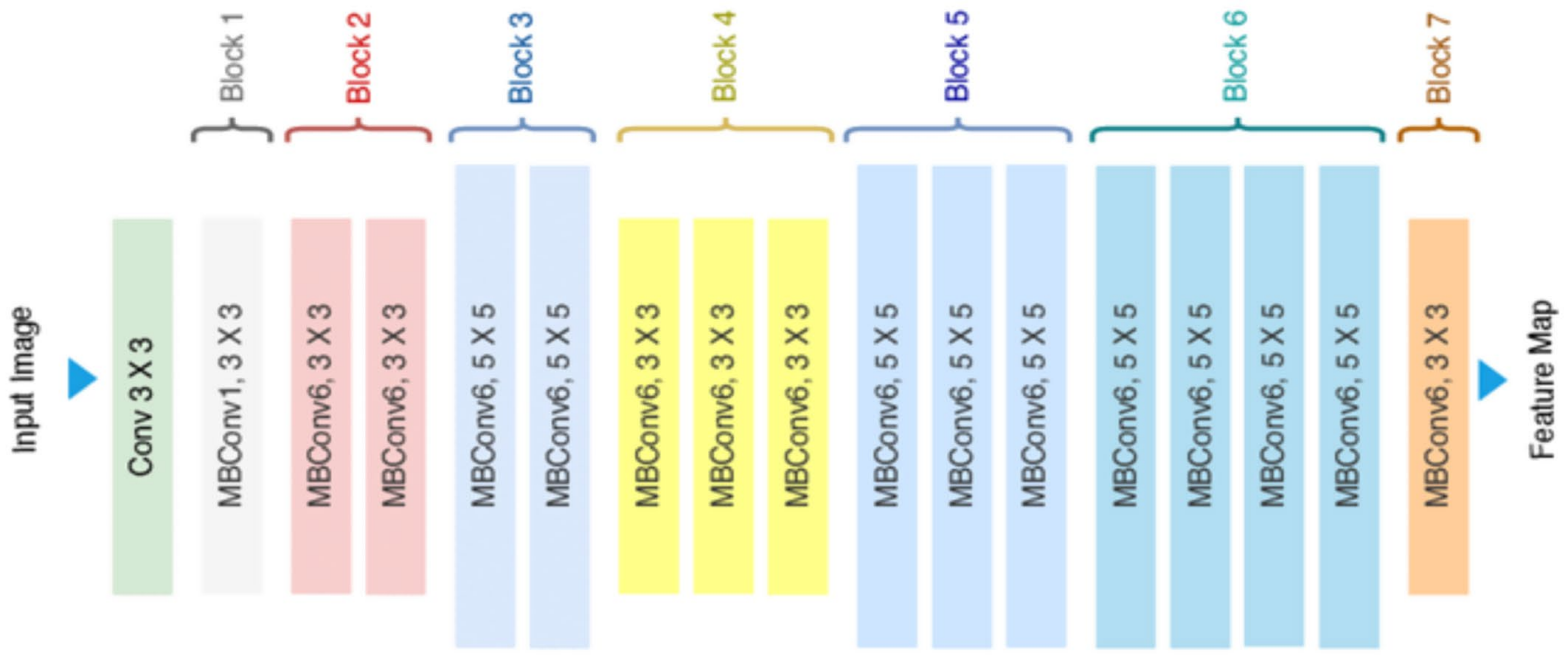
EfficientNetB7 model architecture.

EfficientNetB7 (Figure 11) was used as a high-capacity U-Net encoder, exploiting compound scaling to balance accuracy and computational efficiency. The classification head is replaced with the decoder for pixel-level segmentation. The essential parts of the MBConv blocks along with SE modules boost dynamically the most relevant features enabling the network to focus on discriminative crack patterns in high-resolution images without being computationally prohibitive (Mazni et al., 2024).

### 2.5 Loss function and optimization

For the binary crack segmentation task, the network outputs a single-channel feature map. A sigmoid (Shao and Wang, 2023) activation function is applied pixel-wise at the final layer, mapping each pixel’s raw logit to a probability value in the range [0,1]. This formulation is appropriate for binary segmentation as it treats each pixel’s classification as an independent Bernoulli trial, naturally representing the probability of belonging to the crack class.

The model is optimized using the Binary Cross-Entropy (BCE) loss, which measures the discrepancy between the predicted pixel-wise probabilities ( ${\hat{y}}_{i}$ ) and the ground-truth binary labels ( $y_{i}$ ), defined as:


$$L_{\mathrm{BCE}}=-(\frac{1}{N})\times \Sigma_{i}[\;y_{i}\times \log({\hat{y}}_{i})+(1-y_{i})\times \log(1-{\hat{y}}_{i})]$$ (1)

where N is the total number of pixels. This loss is well-suited for tasks with imbalanced class distributions (such as cracks occupying a small fraction of pixels) because it penalizes errors on both the minority (crack) and majority (background) classes with equal theoretical importance at the pixel level.

Training was performed using the Adam optimizer with a learning rate of 0.01, a batch size of 32, and a fixed schedule of 30 epochs. The model checkpoint yielding the highest validation accuracy was retained for final evaluation to prevent overfitting and ensure robust generalization.

### 2.6 Post-processing

Following binary segmentation, post-processing techniques were applied to extract quantitative morphological metrics (crack length, width, and severity) from the predicted masks.

#### 2.6.1 Skeletonization

The crack skeleton is obtained using thinning algorithms, which reduce cracks to a central axis while preserving connectivity and overall topology. Several methods (Zhang-Suen, Guo-Hall, iterative morphological, fast parallel thinning, MAT, and Distance Transform) were evaluated. The Medial Axis Transform (MAT) was selected because it preserves crack geometry, reduces noise, and accurately defines the central axis—essential for precise length and width measurements.

#### 2.6.2 Crack length measurement

The overall length of cracks is measured using a pixel-based calculation on the skeletonized image produced from segmentation. The algorithm processes each pixel (x,y) in the binary skeleton image, checking a calibration index f(x,y) , which returns 1 for skeleton pixels and 0 otherwise. A point counter n and a total length $L_{C}\;$ are initialized to zero, and the coordinates of the previous skeleton point are stored in previous point.

For each skeleton pixel, the Euclidean distance to the previous skeleton point is computed:


$$\text{displacement}=\sqrt{{\left(x-x_{\text{prev}}\right)}^{2}+{\left(y-y_{\text{prev}}\right)}^{2}}.$$

This displacement is added to the total crack length $L_{C}$ , and previous point is updated to the current pixel. After processing all pixels, $L_{C}\;$ represents the total crack length in pixel units and can be converted to real-world dimensions through calibration. This method ensures continuous and precise measurement along the crack centerline, minimizing errors caused by width variations or noise.

#### 2.6.3 Crack width measurement

Crack width is quantified using the skeletonized image and edge detection. First, the Canny edge detector (thresholds: 100 and 200) is applied to extract crack boundaries. For each skeleton point (x,y) , the nearest edge pixels on the left and right sides of the skeleton are identified, and the Euclidean distances to each edge are computed:


$$d_{\text{left}}=\sqrt{{\left(x_{\text{left}}-x\right)}^{2}+{\left(y_{\text{left}}-y\right)}^{2}},d_{\text{right}}=\sqrt{{\left(x_{\text{right}}-x\right)}^{2}+{\left(y_{\text{right}}-y\right)}^{2}}.$$

The local crack width is calculated as $\text{crack width}=d_{\text{left}}+d_{\text{right}}$ . After processing all skeleton points, statistical measures such as maximum, minimum, and average crack width are derived. The maximum width indicates the most critical portion of the crack, while the average width provides a global severity measure. These pixel-based measurements can be converted to real-world units, ensuring accurate structural assessment.

#### 2.6.4 Crack severity assessment

The severity of cracks has been categorized into three levels--low, medium, and high--in relation to linear and area criteria. Linear based severity is derived from the maximum crack width, whereas area-based severity relates the area damaged to the inspected area. Thresholds were established in collaboration with a construction expert and informed by studies before (Ragnoli et al., 2018) and international guidelines such as ACI 224R-01 (ACI Committee 224, 2001). These criteria, as summarized in Table 3, were deployed on the input sections of 0.6 m × 1.06 m for assessing the crack severity. While high severity cracks indicate unsafe conditions that need immediate attention, medium and low severity cracks indicate less critical damage to the structures.

**Table 3:** Classification criteria for wall crack severity.

Types of cracks	Measure	Severity	Range	Réf.
Linear crack	Width (mm)	Low	X < 10	Ragnoli et al. (2018) and ACI Committee 224 (2001)
		Medium	10 ≤ X < 75	
		High	X ≥ 75	
Area crack	Area (%)	Low	X < 10	
		Medium	10 ≤ X < 25	
		High	X ≥ 25	

##### 2.6.4.1 Linear crack severity

Linear cracks (vertical, horizontal, or diagonal) are assessed based on maximum crack width. Each image (0.6 × 1.06 m, 224 × 224 pixels) corresponds to ~2.68 mm/pixel width and ~4.73 mm/pixel height. Severity is classified as low, moderate, or high using thresholds derived from previous studies (Ragnoli et al., 2018) and international guidelines [ACI 224R-01 (ACI Committee 224, 2001)]. Classification is validated against manually annotated masks.

##### 2.6.4.2 Area crack severity

Area cracks (fissures, spalls, or large surface deterioration) are assessed by the area ratio:


Area Ratio=(Cracked Area/Total Wall Area)×100

Cracked Area = number of pixels belonging to the crack (red pixels in Table 4).

**Table 4:** The results of the UAV’s measurements.

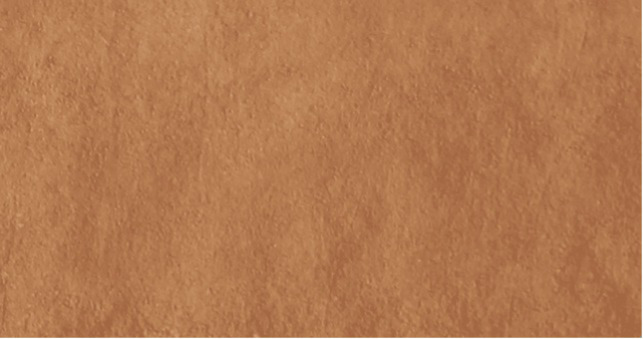	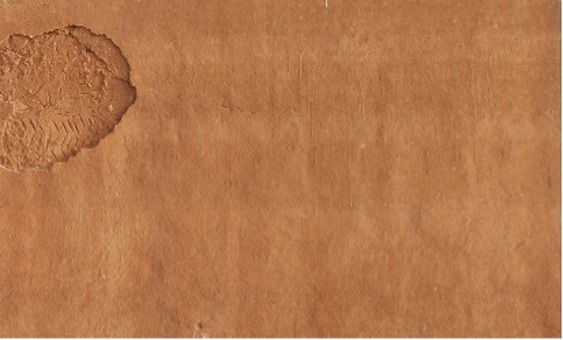	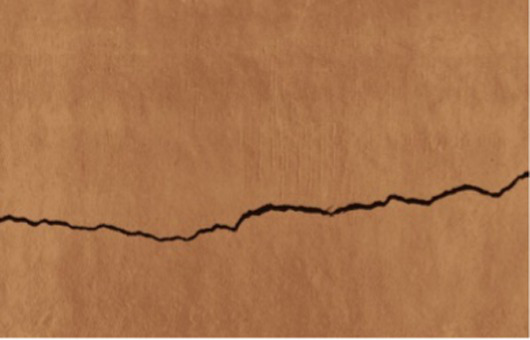
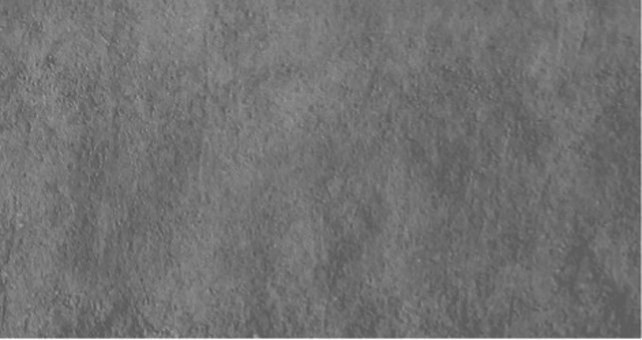	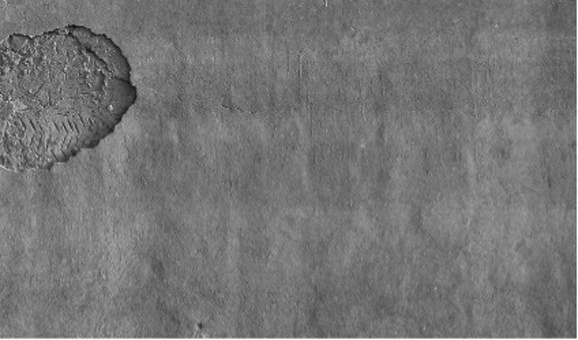	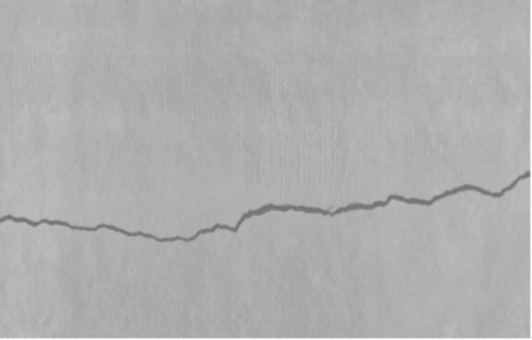
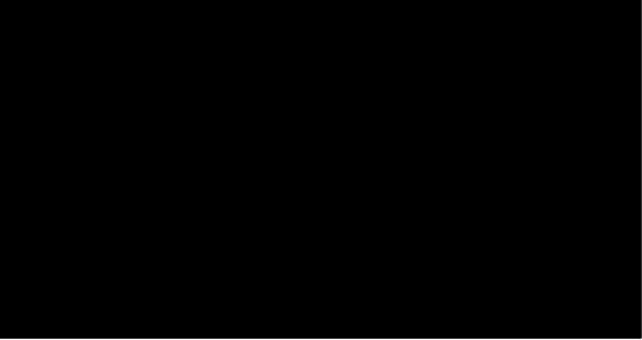	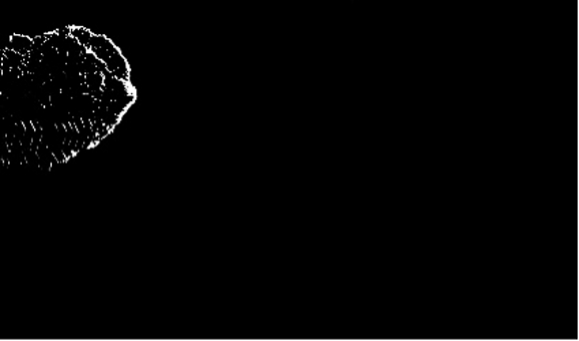	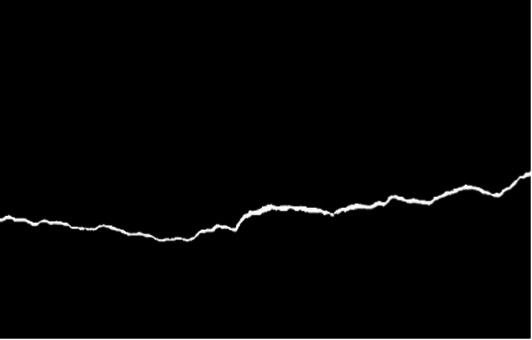
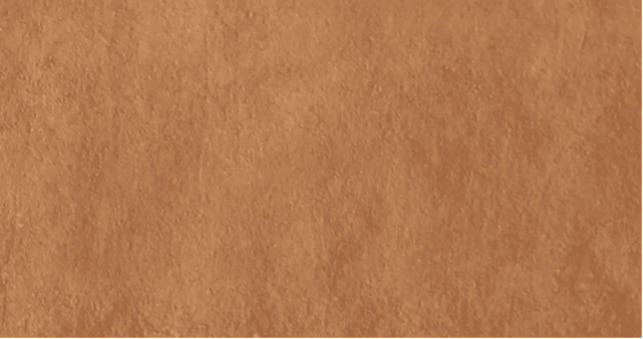	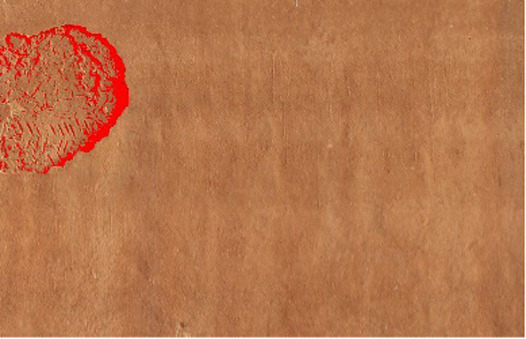	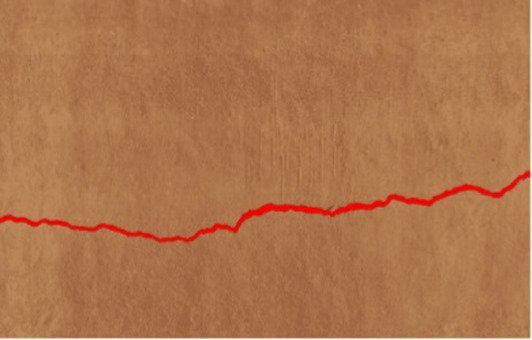
No CRACK Found	CRACK Found Type: Area Crack Total Wall Area (px): 3500 Cracked Area (px): 224 × 224 Area (%): 6.98% Severity: Low (<10%)	CRACK Found Type: Linear Crack Length (px): 393 Mean Width (px): 76.87 Max Width (px): 76.87 Length (mm): 103.98 Avg Width (mm): 20.34 Max Width (mm): 20.34 Severity: Medium

Total Wall Area = total number of pixels in the wall region (the entire image).

Severity is classified as low, medium, or high. Alternatively, the ratio of cracked area to its bounding box can be used, depending on the application. Reliability is checked against manually labeled ground truth.

## 3 Experiments and results

**Figure 12:**
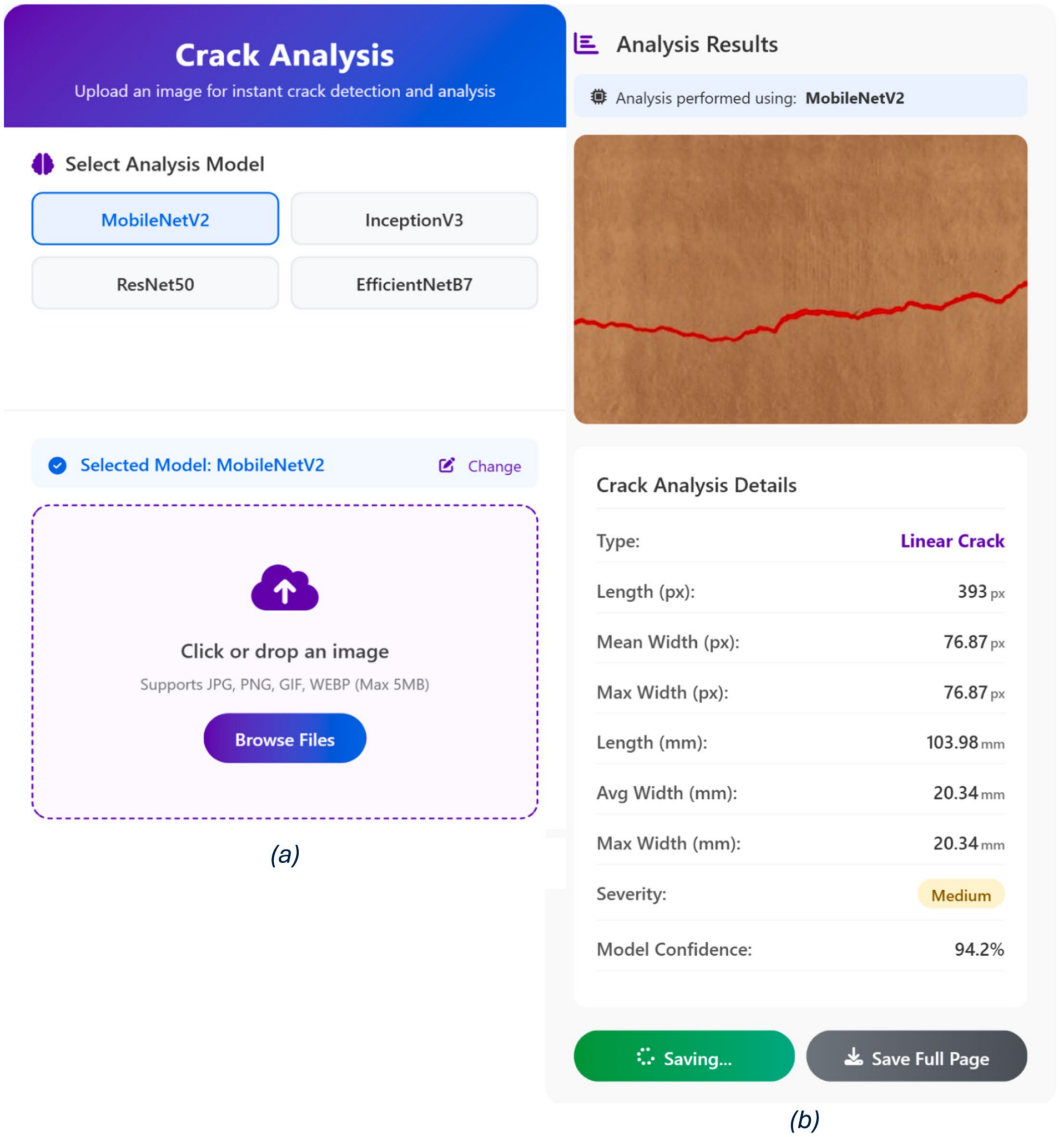
Interface of the proposed web application: **(a)** home screen showing the four selectable deep learning models and drag-and-drop image upload functionality; **(b)** output screen presenting the analysis results for the chosen model.

**Figure 13:**
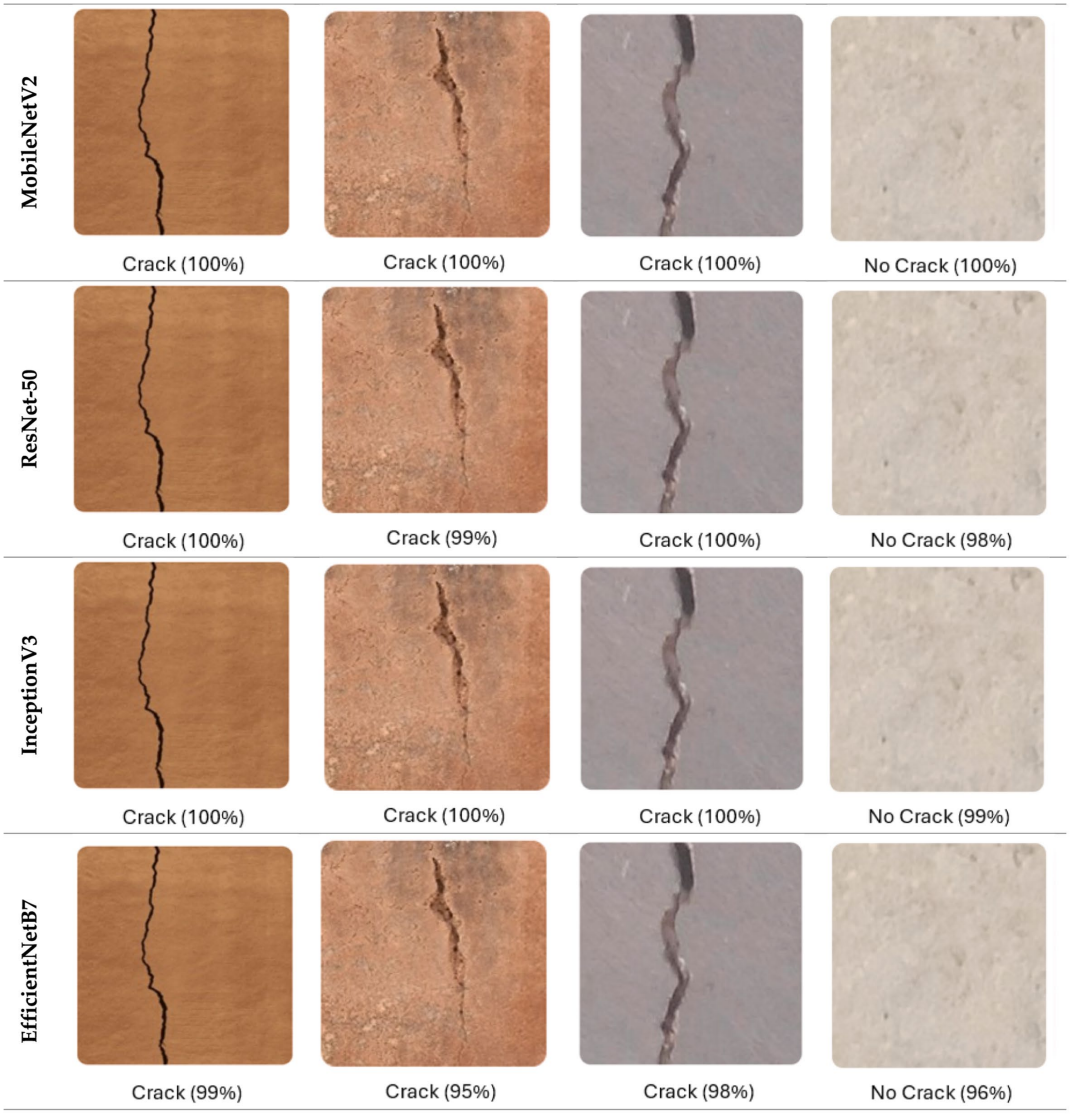
Examples of crack classification results obtained from four CNN models, indicating predicted class and confidence percentage.

To validate the effectiveness of the developed system, tests were conducted on images containing thin and irregular cracks on complex concrete backgrounds. The proposed system is a responsive web application for automated crack detection and analysis, developed in Python (3.10) using the Flask (2.3) framework. Flask enables rapid deployment of deep learning models via RESTful APIs and seamless integration with trained networks. As illustrated in Figure 12, the web interface includes: (a) a home screen for selecting one of four deep learning models (MobileNetV2, ResNet-50, InceptionV3, EfficientNetB7) and uploading images, and (b) an output screen displaying the analysis results. Example classification outputs are shown in Figure 13. The system was tested across various devices and screen sizes to ensure responsiveness and accessibility.

The U-Net architecture was trained using an 80–20% dataset split (≈8,000 training images, ≈2,000 test images) to assess generalization. Training was performed in Python 3.8 with PyTorch, using a batch size of 16 and the Adam optimizer (learning rate 0.001, β1 = 0.9, β2 = 0.999, weight decay 10^−4^). Segmentation used a combination of Dice and Binary Cross-Entropy losses, with a Sigmoid output activation. Models were trained for 100 epochs, with a learning rate reduction factor of 0.1 at epoch 40. Runtime evaluation was performed on an NVIDIA DGX-1 system with dual 20-core Intel Xeon CPUs, 512 GB RAM, and eight Tesla V100 GPUs.

### 3.1 Evaluation metrics

To rigorously assess the proposed deep learning system, two complementary sets of metrics were used: segmentation metrics, which evaluate pixel-level accuracy in localizing cracks, and classification metrics, which assess the model’s ability to correctly label pixels as crack or non-crack. This dual evaluation provides a detailed understanding of model performance at both fine-grained and global levels.

#### 3.1.1 Segmentation metrics

Segmentation metrics measure how accurately the predicted masks match the ground-truth crack regions.

(a) Pixel Accuracy (PA): The proportion of correctly classified pixels over the entire image, expressing the global correctness of the segmentation (Equation 1).


$$\mathrm{PA}=(\sum n_{\mathrm{ii}})/(\sum t_{i})$$ (1)

where *nᵢᵢ* is the number of correctly classified pixels for class *i*, and *tᵢ* is the total number of pixels belonging to class *i*.

(b) Mean Pixel Accuracy (MPA): The accuracy independently for each class and then averages the results. This metric is robust for imbalanced datasets (Equation 2).


$$\mathrm{MPA}=(1/n_{c})\times \sum (n_{\mathrm{ii}}/t_{i})$$ (2)

with *n_c_* representing the number of classes (crack, non-crack).

(c) Intersection over Union (IoU): Measures overlap between predicted and ground-truth regions of class *i*; (Equation 3).


$${\mathrm{IoU}}_{i}=n_{\mathrm{ii}}/(t_{i}+\sum n_{\mathrm{ji}}-n_{\mathrm{ii}})$$ (3)

Higher IoU values indicate more accurate segmentation.

(d) Mean Intersection over Union (MIoU): The averages IoU across all classes, a standard benchmark in semantic segmentation (Equation 4).


$$\text{MIoU}=(1/n_{c})\times \sum {\mathrm{IoU}}_{i}$$ (4)

(e) Dice Coefficient (F1-Score for Segmentation): Evaluates similarity between predicted and reference masks, particularly useful for thin cracks (Equation 5).


Dice=2∣A∩B∣/(∣A∣+∣B∣) (5)

where *A* and *B* are the predicted and ground-truth masks.

#### 3.1.2 Classification metrics

Classification performance was derived from four fundamental quantities in the confusion matrix:

- TP: correctly detected crack pixels
- TN: correctly detected non-crack pixels
- FP: non-crack pixels incorrectly labeled as cracks
- FN: crack pixels incorrectly labeled as non-cracks

(a) Accuracy: Overall proportion of correct predictions (Equation 6).


Accuracy=(TP+TN)/(TP+TN+FP+FN) (6)

(b) Recall/True Positive Rate (TPR): Proportion of actual crack pixels correctly detected (Equation 7).


TPR=TP/(TP+FN) (7)

(c) False Positive Rate (FPR): Proportion of non-crack pixels incorrectly labeled as cracks (Equation 8).


FPR=FP/(FP+TN) (8)

(d) False Negative Rate (FNR): Proportion of crack pixels missed by the model (Equation 9).


FNR=FN/(TP+FN) (9)

(e) True Negative Rate (TNR)/Specificity: Proportion of non-crack pixels correctly identified (Equation 10).


TNR=TN/(TN+FP) (10)

(f) Precision: Proportion of predicted crack pixels that are correct (Equation 11).


Precision=TP/(TP+FP) (11)

(g) F1-Score: Harmonic mean of Precision and Recall, balancing false positives and false negatives (Equation 12).


F1=2TP/(2TP+FP+FN) (12)

### 3.2 Comparative analysis of transfer learning techniques

**Figure 14:**
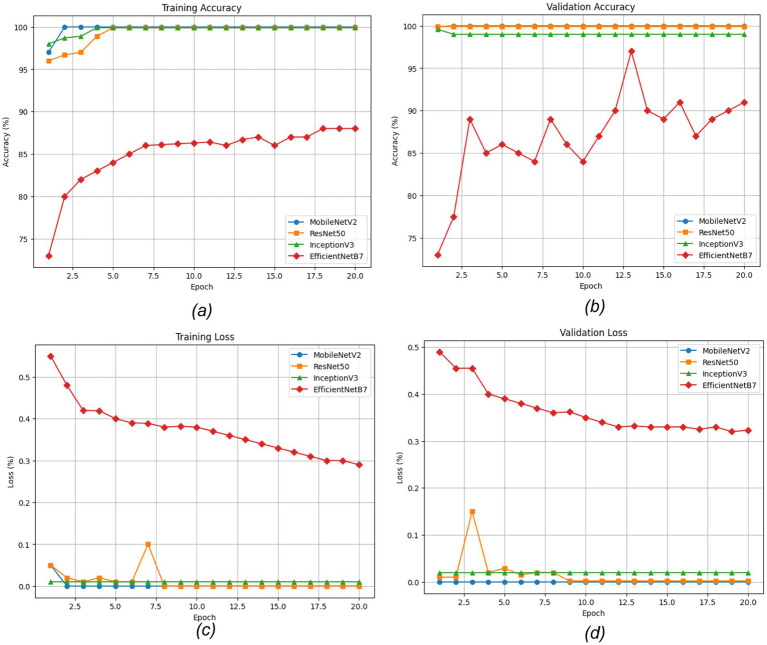
Evaluation of the convergence behavior of the transfer learning models: **(a)** training accuracy, **(b)** validation accuracy, **(c)** training loss, and **(d)** validation loss.

The convergence behavior of the transfer learning models is shown in Figure 14. Figures 14a,b depict the training and validation accuracies, where MobileNetV2, InceptionV3, and ResNet-50 obtained almost perfect scores, and MobileNetV2 showed the best generalization characterized by a small difference in training and validation performance. EfficientNetB7 converged slower and achieved worse accuracies. Figures 14c,d show the training and validation losses. MobileNetV2 and InceptionV3 had their early minima and stable performance thereafter, whereas ResNet-50 showed small oscillations due to temporary overfitting. EfficientNetB7 was observed to have a steady linear decrease in loss. To summarize, this pattern indicates robust learning and generalization in MobileNetV2 and InceptionV3, whereas EfficientNetB7 appeared to converge slowly but consistently decrease in loss.

### 3.3 Crack classification outcomes and performance metrics

Table 5 summarizes the performance of U-Net models with different transfer learning backbones. Evaluated using both segmentation metrics (PA, MPA, Dice, IoU, and mIoU) and classification metrics (Accuracy, Recall, Precision, F1-Score). MobileNetV2 and InceptionV3 achieved the highest and most balanced performance. MobileNetV2 showed excellent generalization (Precision = 99.1%, Recall = 98.2%), while InceptionV3 maintaining robust segmentation (F1-Score = 97.3%, Dice = 97.2%). ResNet-50 was competitive (Accuracy = 96.5%, F1-Score = 95.9%), with minor early overfitting, and EfficientNetB7 had the lowest overall performance, although its higher precision (90.2%) and efficiency make it suitable for low-resource applications.

**Table 5:** Evaluating performance metrics for different pre-trained transfer learning CNN models (%).

Model (%)	PA	MPA	Accuracy	Recall	Precision	F1-score	Dice	IoU	mIoU
MobileNetV2	98.8	98.6	98.7	98.2	99.1	98.6	98.5	97.2	96.8
ResNet-50	96.7	96.2	96.5	95.7	96.2	95.9	95.8	94.1	93.7
InceptionV3	97.6	97.0	97.4	96.8	97.9	97.3	97.2	95.9	95.5
EfficientNetB7	89.3	88.8	89.1	88.4	90.2	89.3	89.0	86.7	86.2

### 3.4 Original per-Class IoU, robustness checks, and confusion matrices

**Figure 15:**
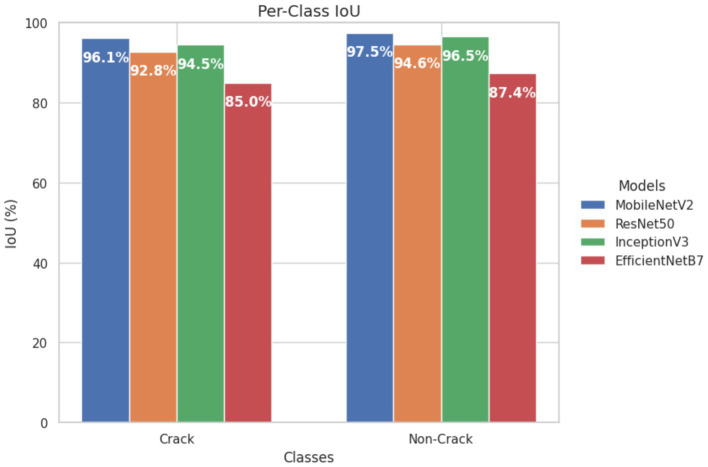
Original per-Class IoU values for different U-Net backbones.

Per-Class IoU: MobileNetV2 achieved the highest IoU values (Crack = 96.1%, non-Crack = 97.5%), followed by InceptionV3 (Crack = 94.5%, non-crack = 96.5%), ResNet-50 (92.8, 94.6%), and EfficientNetB7 (85.0, 87.4%), reflecting relative segmentation capacities (Figure 15).

**Figure 16:**
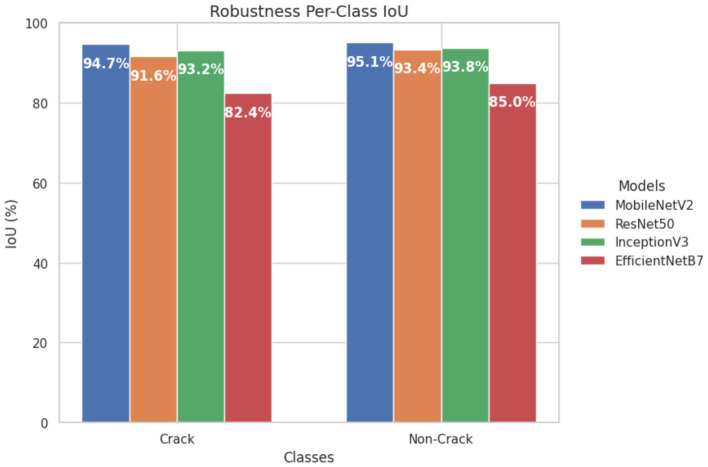
Per-class IoU values under robustness checks.

Robustness checks: Minor perturbations (e.g., noise, rotations) caused small reductions in IoU across all models. MobileNetV2 remained the most robust (Crack = 94.2%, non-Crack = 95.8%), followed by InceptionV3 and ResNet-50, while EfficientNetB7 showed greater sensitivity (Crack = 83.2%, non-crack = 85.5%) (Figure 16).

**Figure 17:**
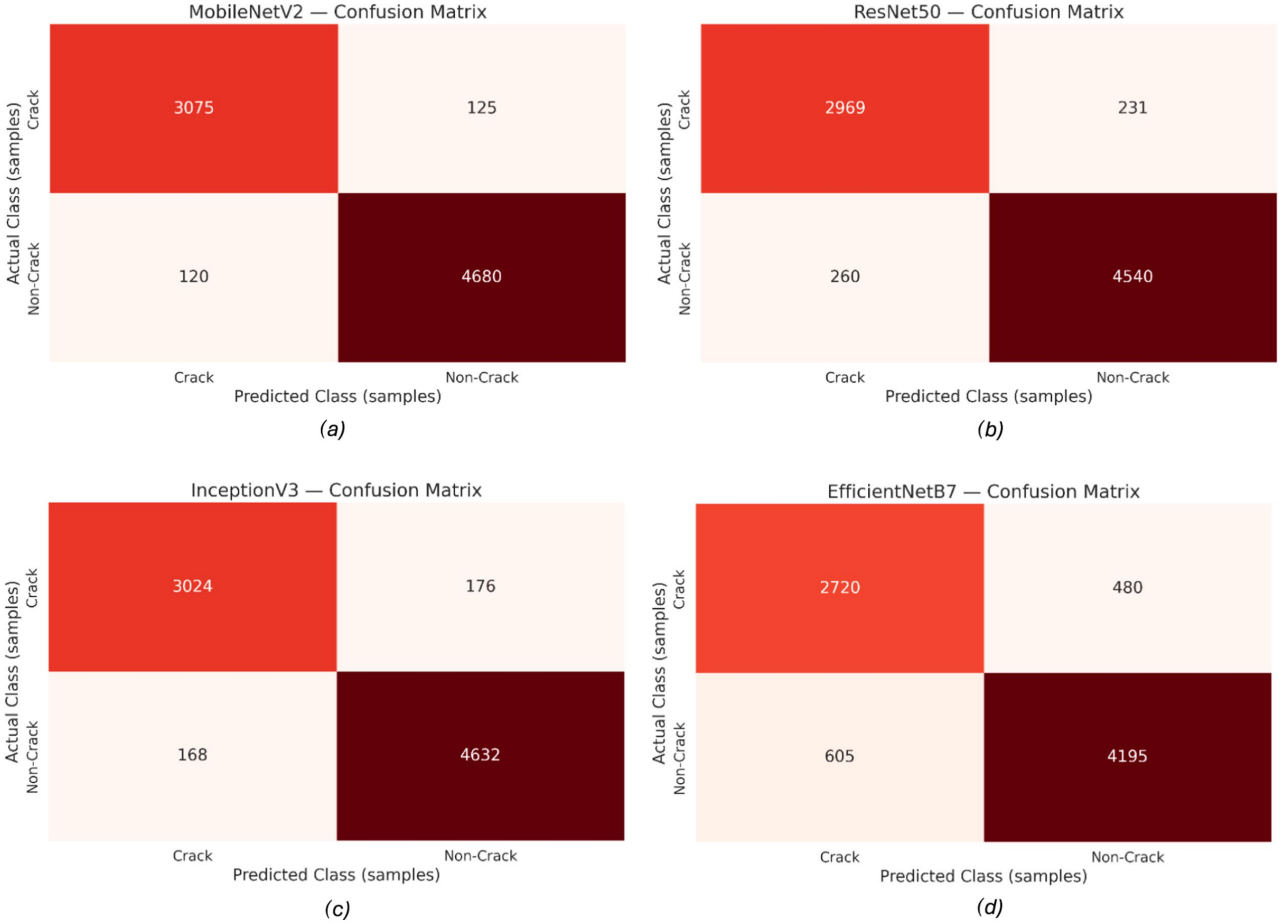
Confusion matrices of the evaluated models: **(a)** MobileNetV2, **(b)** ResNet-50, **(c)** InceptionV3, and **(d)** EfficientNetB7.

Confusion matrices: MobileNetV2 and InceptionV3 had the lowest misclassification rates, correctly identifying the majority of crack and non-crack pixels. EfficientNetB7 exhibited higher counts of false positives and negatives. These trends are visualized in Figure 17 and confirm that MobileNetV2 and InceptionV3 offer both strong pixel-wise segmentation and classification performance, with superior robustness to minor input variations.

## 4 Discussion

The experimental results show that the web-based crack detection system presented here, utilizing U-Net architectures with different options for transfer learning backbones, can achieve very high accuracy when segmenting thin and irregular cracks on intricate concrete surfaces. MobileNetV2 and InceptionV3 consistently outperformed ResNet-50 and EfficientNetB7, offering a balanced trade-off between computational efficiency and generalization (Table 5). MobileNetV2 was the most robust and pixilated in precision, represented by per-Class IoU values of 96.1 percent for cracks and 97.5 percent for non-cracks (Figure 15) with an overall F1-score of 98.6 percent. The convergence behavior (Figure 14) and robustness (Figure 16) checks confirm the stability of these top models, and confusion matrices (Figure 17) and output examples (Figure 13) highlight minimal false positives and good segmentation.

Against other recent studies, our results go beyond many benchmarks. Diakogiannis et al. (2020) report IoU around 95% and Dice scores near 94% using a ResNet-based U-Net; in contrast, our MobileNetV2 backbone achieves promising segmentation quality and generalization. On the other hand, hybrid architectures, such as attention-enhanced U-Nets or Transformers, achieve Dice coefficients that are close to 0.99, but at a much higher computational burden. Classification-focused CNNs achieve high detection accuracy (~97–99%), although without fine-grained pixel-level localization, and object detection frameworks perform fast inference with moderate mIoU (~94–95%) resolutions but fail to capture detailed morphology of cracks (Qayyum et al., 2023; Anusha and Anbarasi, 2025).

On the contrary, the proposed system yields dense, pixel-wise segmentation with robust performance under perturbations and practical interpretability via a web-based interface (Figures 12, 13). Overall, lightweight transfer learning method either meets or surpasses state-of-the-art performance and represents a truly deployable solution for practical applications in the field of structural health monitoring.

## 5 Limitations and assumptions

The proposed technique exhibits good performance subject to given assumptions defining the limits of its operational capability. First, the reported metrics (like MobileNetV2’s 98.7% accuracy) are based on controlled imaging conditions: input images comparable to the training set in terms of resolution, contrast, and lighting conditions, hence devoid of significant shadows, blur, or distortions. Validation has been confined to concrete surfaces, so further adaptations may be necessary to generalize to other materials like asphalt or masonry. Finally, all quantitative outputs such as crack width or area are dependent on segmentation accuracy, which means that any error, however small, at the pixel level will propagate into these measurements. These considerations are critical for placing the system in its practical context.

## 6 Conclusion and future work

This research successfully develops and validates a responsive, web-based system for automated crack detection and quantitative structural health assessment. The primary contributions of this work are fourfold: (1) the implementation and rigorous evaluation of a U-Net architecture enhanced with several state-of-the-art transfer learning backbones (MobileNetV2, InceptionV3, ResNet-50, EfficientNetB7) for precise pixel-wise segmentation; (2) the demonstration of exceptional performance, particularly with MobileNetV2 achieving a mean Intersection over Union (mIoU) of 96.8% and an overall accuracy of 98.7%, establishing a robust benchmark for crack delineation in complex concrete backgrounds; (3) the development of a user-accessible Flask web application that integrates model inference, visualization, and feature extraction into a single, deployable platform; and (4) a comprehensive quantitative analysis using both segmentation (IoU, Dice) and classification (Precision, Recall, F1-Score) metrics, complemented by robustness checks, to provide a holistic evaluation of model performance and generalizability.

The practical value of the system lies in its dual capability for high-accuracy detection and immediate, quantifiable analysis, delivering metrics such as crack width and area through an interface optimized for standard computational resources. This makes the tool both technically sophisticated and operationally practical for engineers and inspectors.

Future work will focus on extending the system to 3D defect quantification by integrating photogrammetry and LiDAR data, enhancing robustness under real-world conditions such as varying lighting and occlusion, expanding detection to a wider range of defect types, and optimizing the framework for real-time, edge-based deployment on-site.

## Data Availability

The datasets presented in this study can be found in online repositories. The names of the repository/repositories and accession number(s) can be found in the article/supplementary material.

## Footnotes

1 The khettara (also known as qanat) is a traditional, earthen water management system (Hayes-Rich et al., 2023).
